# Functionalization of Endohedral Metallofullerenes with Reactive Silicon and Germanium Compounds †

**DOI:** 10.3390/molecules22071179

**Published:** 2017-07-14

**Authors:** Masahiro Kako, Shigeru Nagase, Takeshi Akasaka

**Affiliations:** 1 Department of Engineering Science, The University of Electro-Communications, Chofu 182-8585, Japan; 2 Fukui Institute for Fundamental Chemistry, Kyoto University, Kyoto 606-8103, Japan; 3 Life Science Center of Tsukuba Advanced Research Alliance, University of Tsukuba, Ibaraki 305-8577, Japan; 4 Department of Chemistry, Tokyo Gakugei University, Tokyo 184-8501, Japan; 5 Foundation for Advancement of International Science, Ibaraki 305-0821, Japan; 6 School of Materials Science and Engineering, Huazhong University of Science and Technology, Wuhan 430074, China

**Keywords:** endohedral metallofullerene, silylation, germylation, disilirane, silirane, silylene, digermirane

## Abstract

Exohedral derivatization of endohedral metallofullerenes (EMFs) has been exploited as a useful method for characterizing the structural and chemical properties of EMFs, and for functionalizing them for potential applications. The introduction of heteroatoms, such as electropositive silicon atoms, to fullerene cages is a novel functionalization method that remarkably affects the electronic characteristics of fullerenes. This review comprehensively describes the results of the reactions of monometallofullerene, dimetallofullerene, and trimetallic nitride template EMFs with disilirane, silirane, silylene, and digermirane, which afforded the corresponding silylated and germylated fullerenes. Several examples emphasize that exohedral functionalization regulates the dynamic behaviors of the encapsulated metal atoms and clusters in the fullerene cages. The electronic effects of silyl and germyl groups are represented by comparing the redox properties of silylated and germylated EMFs with those of other EMFs derivatized with carbon-atom-based functional groups.

## 1. Introduction

Endohedral metallofullerenes (EMFs) are attracting great interest because of their unique structures and properties [1,2,3,4,5,6,7,8,9,10,11,12,13,14,15]. Encapsulation of metal atoms and metal clusters inside EMFs results in electron transfer from the inner species to the carbon cages. Results of earlier studies show that EMFs have remarkable electronic properties such as oxidation and reduction potentials. The difference in redox potentials between EMFs and hollow fullerenes is a key factor characterizing the properties and reactivities of EMFs.

Exohedral derivatization of EMFs and empty fullerenes has been explored extensively as an effective method to modify and enhance the characteristics of fullerenes for various nanomaterials science and biomedicine applications [1,2,3,4,5,6,7,8,9,10,11,12,13,14,15]. To date, many reactions have been applied for the exohedral derivatization of EMFs with respect to their use for functional materials. However, the derivatization reactions of EMFs remain sparse compared to those of empty fullerenes. In many cases, the derivatizing organic groups are bonded covalently to the carbon cages of EMFs through C–C bonds. Therefore, derivatization that exploits the heteroatom properties is expected to contribute to the development of EMFs as functional materials.

**Figure 1:**
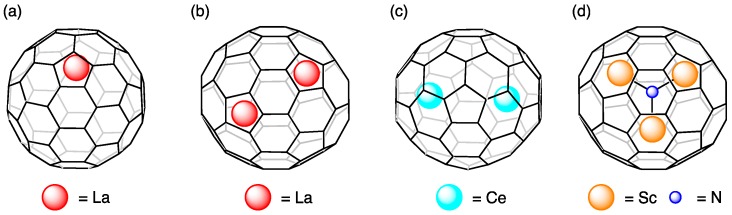
Structures of (**a**) La@*C*_2*v*_-C_82_; (**b**) La_2_@*I_h_*-C_80_; (**c**) Ce_2_@*D*_3*h*_-C_78_; and (**d**) Sc_3_N@*I_h_*-C_80_.

In this context, derivatization of fullerenes using silicon and germanium atoms is an attractive method for modifying the electronic properties of fullerenes because of the electron-donating effects of silyl and germyl groups [16,17,18,19]. We found that the reactions of empty fullerenes with reactive silicon and germanium compounds afforded the corresponding fullerene derivatives [20,21,22,23,24,25,26,27,28,29,30,31,32]. These results prompted us to investigate properties of EMFs by examining their reactivities toward silicon and germanium compounds. The combination of fullerenes and silicon and germanium compounds provide a basis for the production of novel functional materials for electronic device, as mentioned in the section on potential applications. This review presents a summary of our results of silylation and germylation reactions as part of our continuing investigation of the chemical functionalization of EMFs [1,4,5,6,7,8,11,12,15]. We demonstrate that the introduction of silicon and germanium atoms markedly perturbs the electronic properties of fullerenes because of the electron-donating effects of these atoms. More specifically, derivatization of EMFs using reactive compounds containing heavier group 14 elements, such as silylene (silicon analog of carbene), disilirane (disilacyclopropane), silirane (silacyclopropane), and digermirane (digermacyclopropane) will be outlined. For several silylated and germylated derivatives of EMFs, the electronic properties are discussed in comparison with reference compounds that possess the same addition patterns because the redox behaviors of derivatized EMFs depend on the regiochemistry of the addends on the carbon cages. We also describe how derivatization with organosilicon compounds influences the behaviors of encapsulated metal atoms. In this review, we will focus on the functionalization of the following EMFs: La@*C*_2*v*_-C_82_, La_2_@*I_h_*-C_80_, Ce_2_@*I_h_*-C_80_, Ce_2_@*D*_3*h*_-C_78_, Sc_3_N@*I_h_*-C_80_, and Lu_3_N@*I_h_*-C_80_ (Figure 1).

## 2. Comparative Studies of Reactions of EMFs with Disilirane

**Scheme 1:**
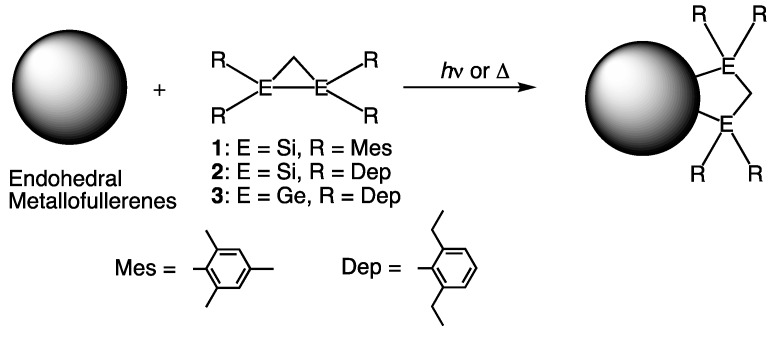
Synthesis of disilirane and digermirane adducts of EMFs.

In 1995, the first chemical derivatization of EMFs was achieved by the photochemical reaction of La@*C*_2*v*_-C_82_ and disilirane **1** [33]. It is particularly interesting that La@*C*_2*v*_-C_82_ reacted both thermally and photochemically with **1** to afford the corresponding adduct, although the empty fullerenes C_60_ and C_70_ did not react with **1** under thermal reactions in the absence of photoirradiation [21,23]. This result can be reasonably explained by the higher electron-accepting ability of La@*C*_2*v*_-C_82_ than that of empty fullerenes. Since this discovery, the reactivities of various EMFs have been investigated using **1** as a chemical probe (Scheme 1). Digermirane **3** reacted with La@*C*_2*v*_-C_82_ showing higher thermal reactivity than **1**, which results from the enhanced electron-donating property of **3** [34].

**Figure 2:**
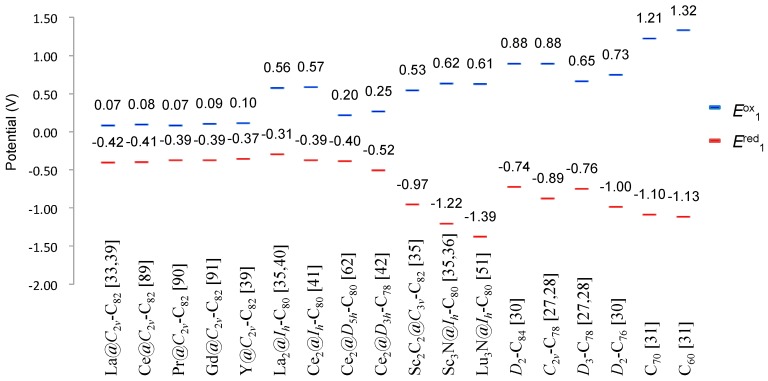
Redox potentials of EMFs and empty fullerenes. Values are in V relative to the ferrocene/ferrocenium couple (Fc/Fc^+^).

Redox potentials of selected EMFs and empty fullerenes are shown in Figure 2. Values of the first reduction (denoted as *E*^red^_1_) potentials are regarded as an important index for the reactivities toward **1** because fullerenes are believed to function as electron acceptors toward **1**, which is a good electron donor [15]. All the fullerenes shown in Figure 2 reacted with **1** by photoirradiation to afford the corresponding adducts. However, the EMFs and empty fullerenes with lower *E*^red^_1_ potentials did not undergo addition reactions under thermal conditions at 80 °C [15]. For example, La_2_@*I_h_*-C_80_ reacted with **1** both photochemically and thermally [35], although Sc_3_N@*I_h_*-C_80_ was found to be reactive only under photolytic conditions [36,37], although the first oxidation (denoted as *E*°^x^_1_) potentials of these EMFs are similar. Therefore, the higher thermal reactivity of La_2_@*I_h_*-C_80_ toward **1** might be attributed to its good electron acceptor properties.

It is noteworthy that the reactivities of EMFs were tunable by chemical oxidation or reduction [38]. Oxidation of La@*C*_2*v*_-C_82_ by (*p*-BrC_6_H_4_)_3_N^+^SbCl_6_^−^ produced the corresponding cation, [La@*C*_2*v*_-C_82_]^+^SbCl_6_^−^, which reacted with **1** even at room temperature. In contrast, the addition of NaSCH_3_ to La@*C*_2*v*_-C_82_ afforded the corresponding anionic species, Na^+^[La@*C*_2*v*_-C_82_]^−^, which was found to be inert to **1**. These results demonstrate that the electrophilicity of EMFs plays an important role in controlling their reactivities toward **1**.

## 3. Reactions of La@*C*_2*v*_-C_82_ with Disilirane 1

Figure 2 shows that La@*C*_2*v*_-C_82_ is highly reactive toward **1** because of its good electron-accepting ability. The molecular symmetry of La@*C*_2*v*_-C_82_, which possesses 24 non-equivalent carbon atoms in the *C*_2*v*_-C_82_ cage, is low. Therefore, addition reactions of La@*C*_2*v*_-C_82_ might produce multiple structural isomers. In fact, thermal reactions of La@*C*_2*v*_-C_82_ with **1** afforded at least six isomers of the mono-adducts based on analysis of the EPR spectrum of the reaction mixture [33]. A similar result was obtained for the thermal addition of digermirane **3** [34], indicating that the addition reactions of **1** and **3** to La@*C*_2*v*_-C_82_ were not regioselective.

The major isomer of the adducts derived from the reaction of La@*C*_2*v*_-C_82_ with **1** was isolated using HPLC separation [39]. The EPR spectrum of this isomer showed only one set of an octet hyperfine that was smaller than that of pristine La@*C*_2*v*_-C_82_. The addition of **1** also caused a change in the absorption spectrum, suggesting that the π-conjugation of the carbon cage was altered. Although the structures of the disilirane adducts of La@*C*_2*v*_-C_82_ have not been determined, their redox properties were obtained from electrochemical measurements. The results show that the *E*°^x^_1_ and *E*^red^_1_ potentials of the disilirane adducts of La@*C*_2*v*_-C_82_ were shifted cathodically, respectively, by 140 mV and 80 mV. These cathodic shifts of the redox potentials of silylated derivatives of EMFs are reasonably explained by the electron-donating effects of the silyl group, including *σ*–*π* conjugation between the C–Si *σ*-orbitals and the *π*-orbitals of the fullerene cages [16,17,18,19]. The reaction of Y@*C*_2*v*_-C_82_ with **1** afforded two isomeric products as the 1:1 adducts, for which the cathodic shifts of redox potentials were observed compared to those of pristine Y@*C*_2*v*_-C_82_ [39].

## 4. Reactions of La_2_@*I_h_*-C_80_ and Related EMFs with Disiliranes 1 and 2

Due to the symmetrical structure of the *I_h_*-C_80_ cage, the number of isomers is limited in the addition reactions of La_2_@*I_h_*-C_80_. Only two non-equivalent carbon atoms exist in the *I_h_*-C_80_ cage: one is shared by one five-membered ring and two six-membered rings (denoted as the [5,6,6]-ring junction); the other is shared by three six-membered rings (denoted as the [6,6,6]-ring junction). The *I_h_*-C_80_ cage also has two non-equivalent C–C bonds: one is shared by one five-membered ring and one six-membered ring (denoted as the [5,6]-ring junction); the other is shared by two six-membered rings (denoted as the [6,6]-ring junction).

**Scheme 2:**
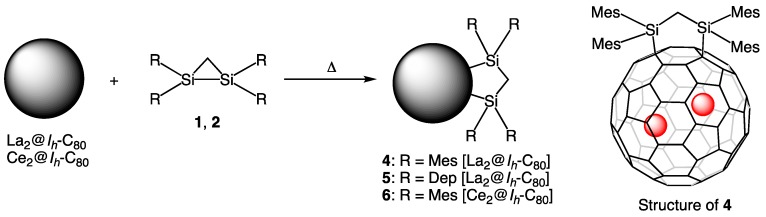
Synthesis of disilirane adducts of M_2_@*I_h_*-C_80_ (M = La, Ce).

Heating a toluene solution of La_2_@*I_h_*-C_80_ and disilirane **2** at 80 °C afforded the corresponding 1:1 adduct **5** as the sole product [40] (Scheme 2). The NMR and X-ray crystallographic analyses established the structures of **5** as the 1,4-adduct, indicating that the silyl groups are bonded to the [5,6,6]-junctions of the *I_h_*-C_80_ cage. In the crystal structure, the two encapsulated metal atoms were located close to the two six-membered rings on the equator of the cage. The reaction of La_2_@*I_h_*-C_80_ and **1** produced a similar adduct **4**. The corresponding adduct of Ce_2_@*I_h_*-C_80_ **6** was also obtained by a similar thermal reaction with **1**, showing the same 1,4-addition pattern [41] (Scheme 2).

In this review, the parent EMFs are shown respectively in brackets.

**Scheme 3:**
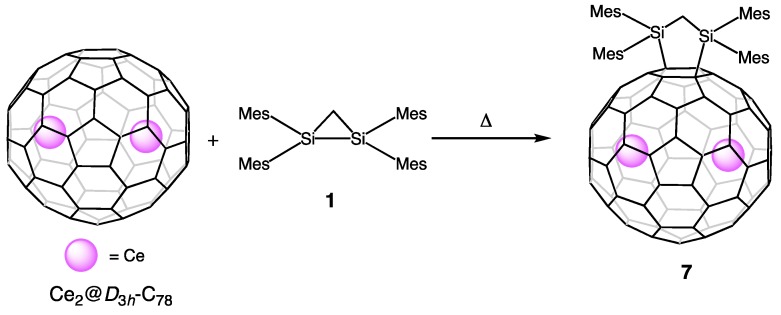
Synthesis of a disilirane adduct of Ce_2_@*D*_3*h*_-C_78_.

Scheme 3 shows that Ce_2_@*D*_3*h*_-C_78_ also reacts readily with disilirane **1** to produce the corresponding 1,4-adduct **7** [42]. Based on X-ray crystallographic studies, the Ce atoms were found to be located at two positions facing the six-membered rings on the *C*_3_ axis of the *D*_3*h*_-C_78_ cage. In the paramagnetic ^13^C-NMR spectral analysis, the observed carbon signals were more temperature-dependent than those of pristine Ce_2_@*D*_3*h*_-C_78_. Therefore, the two Ce atoms in **7** likely interact with the C_78_ cage more firmly than those in pristine Ce_2_@*D*_3*h*_-C_78_ [42].

## 5. Reactions of La_2_@*I_h_*-C_80_ with Silirane 8

**Scheme 4:**
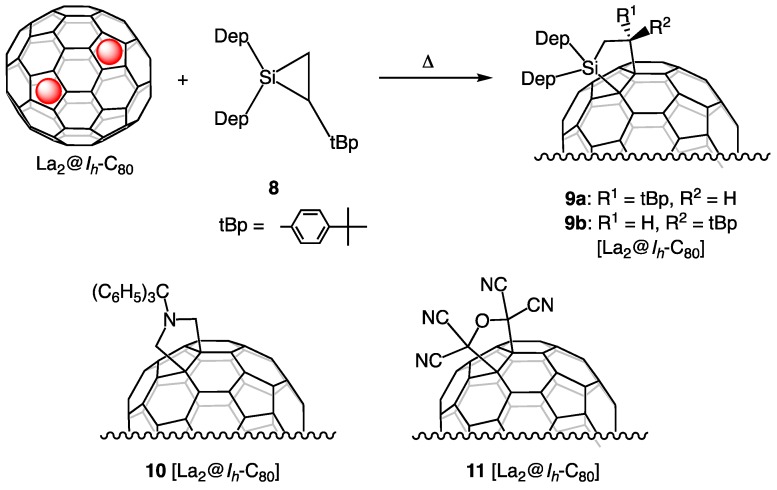
Synthesis of silirane adducts of La_2_@*I_h_*-C_80_. Partial structures are shown for **9a**, **9b**, **10** and **11**.

Scheme 4 shows that La_2_@*I_h_*-C_80_ also reacts with silirane **8** under thermal conditions to afford the corresponding 1,2-carbosilylated derivatives **9a** and **9b** [43]. Structural analyses, including X-ray crystallographic measurements, determined **9a** and **9b** to be a pair of diastereomers of the [5,6]-adducts. Furthermore, electrochemical measurements revealed that carbosilylation slightly altered the electronic properties of La_2_@*I_h_*-C_80_. Results of X-ray crystallographic analysis showed that the crystal structure of **9a** has eight sites for the La atoms because of disorder [43]. Theoretical calculations of adducts of La_2_@*I_h_*-C_80_ and **8** indicated that changing the La atom positions resulted in the differences in relative energies within the range of 0.85–5.89 kcal mol^−1^. These small energy differences suggest the movability of the La atoms inside the cage [43]. The La atoms sites are arranged along the ten contiguously fused six-membered rings that constitute part of the *I_h_*-C_80_ cage.

**Figure 3:**
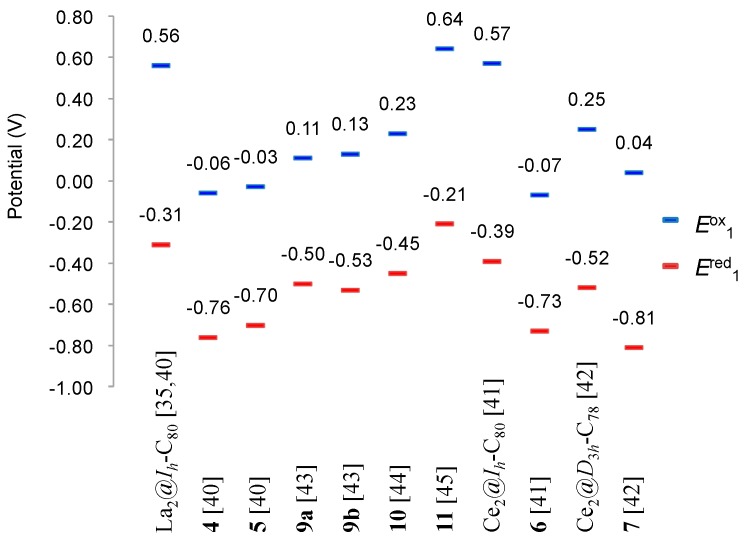
Redox potentials (in V vs. Fc/Fc^+^) of pristine and derivatized dimetallofullerenes.

Figure 3 presents the redox potentials of pristine and derivatized dimetallofullerenes. Remarkable cathodic shifts of both the *E*°^x^_1_ and *E*^red^_1_ potentials were observed for the silylated derivatives **4**, **5**, **9a** and **9b** relative to that of pristine La_2_@*I_h_*-C_80_. Results also confirmed that the redox potentials shifted cathodically as the number of silicon atoms introduced into La_2_@*I_h_*-C_80_ increased as shown by those of **4**, **5**, **9a**, **9b** and **10** [40,43,44]. The electronic effects of the addends are represented more exactly by comparing the [5,6]-carbosilylated adducts **9a**, **9b**, the [5,6]-pyrrolidino adduct **10** [44], and the [5,6]-tetracyanoethylene oxide (TCNEO) adduct **11** [45], which have the same addition patterns. As expected from the effects of the electron-donating silyl group and electron-withdrawing TCNEO moieties, the redox potentials of **9a**/**9b** and **11** shifted, respectively, to more negative and more positive potentials than those of reference compound **10**. Similar cathodic shifts of the redox potentials were observed for **6** and **7**, although the shift of *E*°^x^_1_ potential of **7** was somewhat smaller than expected for a disilirane adduct.

## 6. Reactions of Sc_3_N@*I_h_*-C_80_ with Disilirane 1

**Scheme 5:**
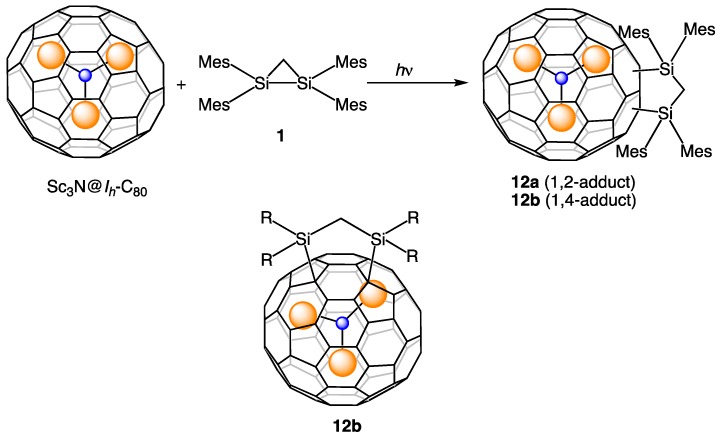
Synthesis of disilirane adducts of Sc_3_N@*I_h_*-C_80_.

Details of the structures and physical properties of silylated derivatives of Sc_3_N@*I_h_*-C_80_, the most abundant trimetallic nitride template (TNT) fullerene, have been reported [37]. Irradiation of disilirane **1** and Sc_3_N@*I_h_*-C_80_ in a mixed solvent of toluene/1,3,5-trichlorobenzene (3/1) produced the corresponding adducts, which were subsequently purified using HPLC to afford a mixture of two adducts **12a** and **12b** in a ratio of approximately 3:2 (Scheme 5). Although **12a** and **12b** could not be isolated, the NMR spectra of the mixture suggested that **12a** and **12b** consisted, respectively, of a 1,2-adduct at the [5,6]-junction, and a 1,4-adduct at the [5,6,6]-junctions on the *I_h_*-C_80_ cage. It is particularly interesting that when a mixture of **12a** and **12b** was heated in *o*-dichlorobenzene (ODCB) at 353 K, facile isomerization of **12a** to **12b** occurred, which suggests that **12b** is thermodynamically more stable than **12a**. The structure of **12b** was confirmed by X-ray crystallographic analysis to be the 1,4-adduct. Results also indicate the location of the encapsulated Sc_3_N cluster, which is oriented so that the Y-shaped Sc_3_N cluster straddles the addition site. Furthermore, theoretical calculations suggest that the free circular motion of the Sc_3_N cluster is restricted in **12b**. Similar addition patterns were reported for the 1,4-adducts, Sc_3_N@*I_h_*-C_80_(CF_3_)_2_ [46] and Sc_3_N@*I_h_*-C_80_(CH_2_C_6_H_5_)_2_ [47], which were synthesized, respectively, by radical addition reactions of CF_3_ and CH_2_C_6_H_5_ radicals.

**Figure 4:**
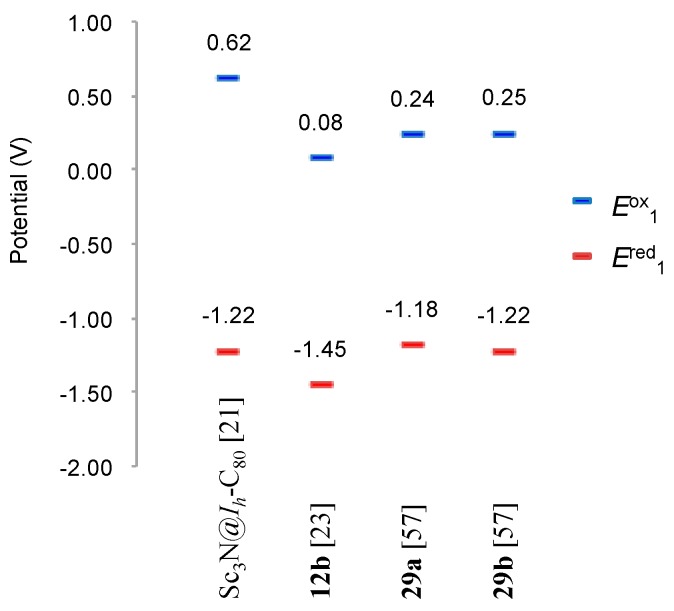
Redox potentials (in V vs. Fc/Fc^+^) of pristine and derivatized Sc_3_N@*I_h_*-C_80_.

The *E*°^x^_1_ and *E*^red^_1_ potentials of **12b** shifted remarkably to negative values by 540 and 230 mV, respectively, compared to those of pristine Sc_3_N@*I_h_*-C_80_, reflecting the addition of two silyl groups (Figure 4). These shifts of the redox potentials correspond to changes in the lowest unoccupied molecular orbital (LUMO) and highest occupied molecular orbital (HOMO) levels according to theoretical calculations [37]. The HOMO and LUMO levels of **12b** are higher than those of pristine Sc_3_N@*I_h_*-C_80_. In addition, the HOMO–LUMO energy gap of **12b** is smaller than that of Sc_3_N@*I_h_*-C_80_. For comparison, the *E*°^x^_1_ and *E*^red^_1_ potentials of Sc_3_N@*I_h_*-C_80_(CF_3_)_2_ were reported as +0.47 V and −1.16 V versus the ferrocene/ferrocenium couple (Fc/Fc^+^), respectively, in agreement with the electron-withdrawing effects of the CF_3_ groups [46].

## 7. Reactions of Lu_3_N@*I_h_*-C_80_ with Silylene

In recent years, among the TNT EMFs, Lu_3_N@*I_h_*-C_80_ has emerged as a suitable acceptor for organic solar cells because the LUMO level of Lu_3_N@*I_h_*-C_80_ is higher than that of C_60_ and other TNT EMFs, M_3_N@*I_h_*-C_80_ (M = Sc, Y, etc.) [48,49]. Indeed, methano-bridged derivatives of Lu_3_N@*I_h_*-C_80_ have been synthesized for application in organic photovoltaic (OPV) devices, which show increases in open circuit voltage and higher efficiencies. For the application of EMFs to OPV devices, the silylation reaction is attractive to adjust the LUMO levels of the EMFs. These results prompted us to investigate the synthesis and properties of organosilicon derivatives of Lu_3_N@*I_h_*-C_80_.

**Scheme 6:**
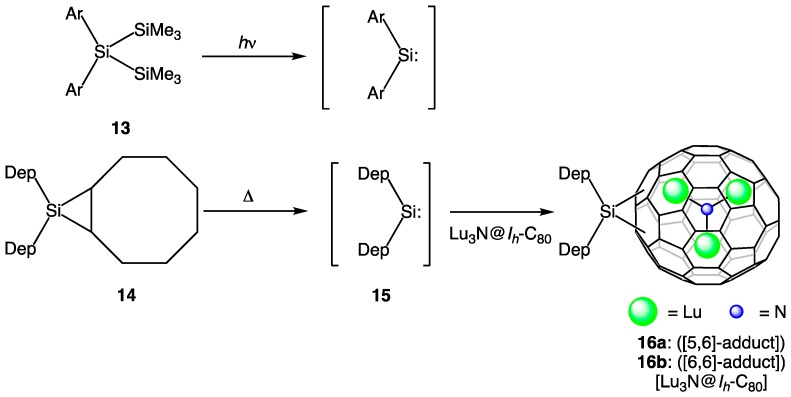
Synthesis of silylene adducts of Lu_3_N@*I_h_*-C_80_.

Previously, the addition of silylene to C_60_ was achieved by photochemical reactions using trisilane compounds **13** [50] as precursors of silylenes (Scheme 6). However, this reaction requires short-wavelength ultraviolet irradiation using low-pressure mercury lamps. When a solution of Lu_3_N@*I_h_*-C_80_ and **13** was photolyzed via ultraviolet irradiation, no silylene adduct was obtained, whereas Lu_3_N@*I_h_*-C_80_ was consumed. This result indicates that ultraviolet irradiation is unsuitable for silylene addition reactions of EMFs. Therefore, we synthesized bicyclic silirane **14** as an alternative silylene precursor, which extrudes **15** under thermal reaction conditions [51]. An ODCB solution of **14** and Lu_3_N@*I_h_*-C_80_ was heated in the dark to afford two silylene mono-adducts **16a** and **16b** (Scheme 6). Based on NMR analysis, the structures of **16a** and **16b** were determined respectively to be [5,6]- and [6,6]-adducts. Furthermore, the fulleroid structure of **16a** was established via X-ray crystallographic analysis. Adducts **16a** and **16b** were sensitive to ambient light. They decomposed gradually to afford pristine Lu_3_N@*I_h_*-C_80_. In addition, the isomerization of **16b** to **16a** was also observed upon exposure to ambient light. In contrast, in the addition of silylene to Sc_3_N@*I_h_*-C_80_ using **14** under the same conditions, the reaction was not efficient. It afforded a poor product yield, preventing further structural elucidation. Based on the electrophilic character of silylenes, the HOMOs of M_3_N@*I_h_*-C_80_ (M = Sc and Lu) might play an important role in their different reactivities toward **15**.

**Figure 5:**
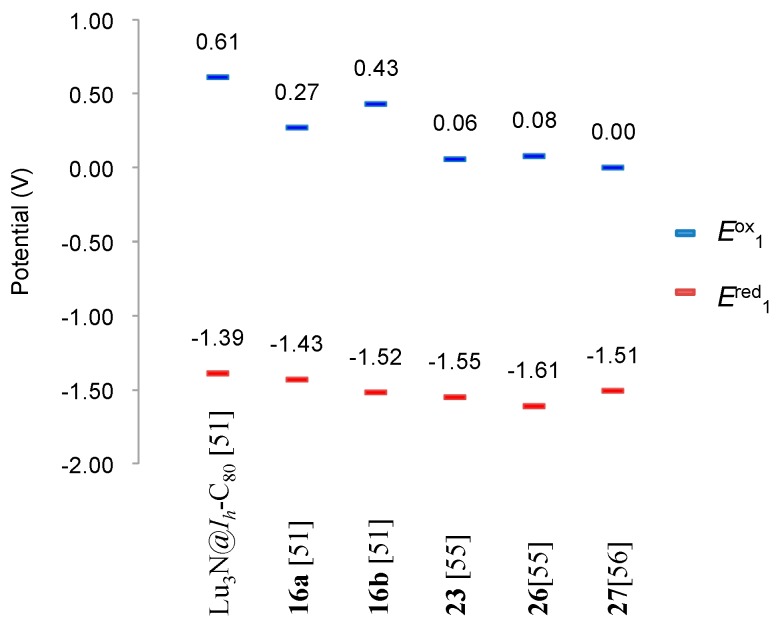
Redox potentials (in V vs. Fc/Fc^+^) of pristine and derivatized Lu_3_N@*I_h_*-C_80_.

**Figure 6:**
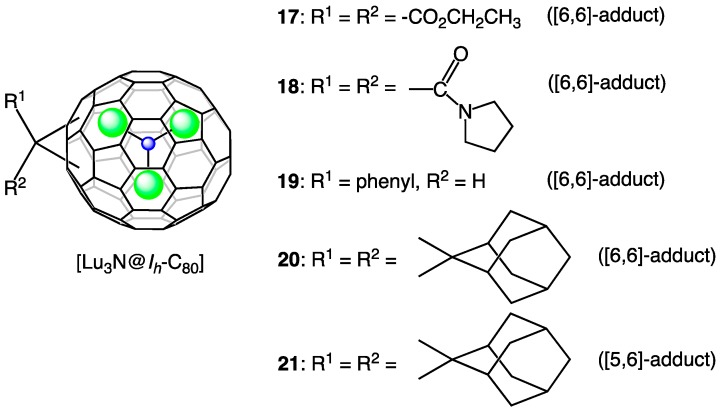
Structures of **17**–**21**.

Electrochemical measurements yielded the voltammograms of **16a** and **16b**, which showed irreversible oxidation and reduction processes. The formation of pristine Lu_3_N@*I_h_*-C_80_ was observed from electrochemical measurements [51]. The *E*°^x^_1_ potentials of **16a** and **16b** shifted toward more negative values by 340 and 180 mV, respectively, compared with those of Lu_3_N@*I_h_*-C_80_ (Figure 5). However, the cathodic shifts of the *E*^red^_1_ potentials of **16a** and **16b** were observed to be 40 and 130 mV, respectively, relative to that of Lu_3_N@*I_h_*-C_80_. As observed for **16a** and **16b**, the redox behaviors of derivatized EMFs depend on the addition pattern on the carbon cages. The redox potentials of **16a** and **16b** were also compared with those of the [6,6]-Bingel–Hirsch derivatives **17** [52], **18** [52], **19** [53], the [6,6]-carbene adduct **20** [54], and the [5,6]-carbene adduct **21** [54] (Figure 6). The *E*°^x^_1_ values of the **17**–**21** were shifted less cathodically than those of **16a** and **16b**, indicating that the addition of silylenes raises the HOMO levels of the products. In addition, the cathodic shifts of the *E*^red^_1_ values of the [6,6]-adduct **16b** and the [5,6]-adduct **16a** are 130 and 40 mV, respectively, compared to that of Lu_3_N@*I_h_*-C_80_. It is noteworthy that those shifts of **17**–**19** ([6,6]-adducts) are 30–160 mV, while that of **21** ([5,6]-adduct) is 70 mV [52,53,54].

## 8. Reactions of Lu_3_N@*I_h_*-C_80_ with Disilirane and Digermirane

**Scheme 7:**
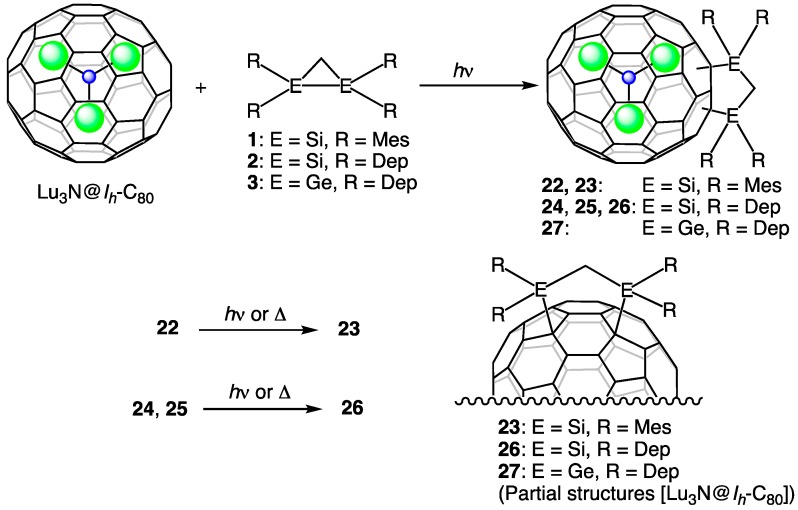
Synthesis of disilirane and digermirane adducts of Lu_3_N@*I_h_*-C_80_, and isomerization reactions of **22**, **24** and **25**.

The electronic effects of silylene on the redox properties of EMFs were found to be moderate, as observed for **16a** and **16b**. Therefore, the reactions between Lu_3_N@*I_h_*-C_80_ and disiliranes **1** and **2** were investigated to evaluate the electronic effects of bis-silylation on pristine Lu_3_N@*I_h_*-C_80_ [55]. Photoreaction of Lu_3_N@*I_h_*-C_80_ and disilirane **1** (or **2**) was conducted in a mixed solvent of ODCB/toluene using halogen–tungsten lamps. As shown in Scheme 7, the reaction of **1** afforded **22** and **23**, whereas that of **2** afforded **24**, **25**, and **26**, respectively, as 1:1 adducts of Lu_3_N@*I_h_*-C_80_ and disiliranes. In addition, **22**, **24**, and **25** were found to be unstable; **22** readily isomerized to **23**, whereas **24** and **25** isomerized to **26**. Therefore, elucidation of the structures and physical properties of **22**, **24**, and **25** has been hitherto unsuccessful. The stable adducts **23** and **26** were determined to be the 1,4-adducts on the [5,6,6]-junctions of the *I_h_*-C_80_ cage via single X-ray crystallographic analysis [56].

Furthermore, the photoreaction of Lu_3_N@*I_h_*-C_80_ with digermirane **3** was investigated to compare the electronic properties of the germylated and silylated derivatives [56]. Due to the higher orbital energy levels of C–Ge bonds compared to those of C–Si bonds, it is expected that germylation will perturb the electronic properties of fullerenes effectively. It is particularly interesting that the photoreaction of Lu_3_N@*I_h_*-C_80_ with **3** proceeded faster than with **2** to afford the corresponding 1,4-adduct **27** under the same reaction conditions (Scheme 7). The structure of **27** was also confirmed using X-ray crystallographic analysis. Results of our earlier study demonstrate that C_60_ functions as an electron acceptor in the photoreactions with **1** [25]. Therefore, a similar electron donor–acceptor interaction is presumed to occur in the case of Lu_3_N@*I_h_*-C_80_. In fact, the high electron donating property of **3** is explained respectively based on the oxidation peak potentials of **2** (+0.54 V) and **3** (+0.45 V) relative to the Fc/Fc^+^ couple. This result was also supported by comparison of the HOMO levels of **2** and **3** obtained using theoretical calculations. Furthermore, the calculated structures of **2** and **3** indicate that the Ge–Ge and Ge–C bonds of **3** are slightly longer, respectively, than the Si–Si and Si–C bonds of **2**. As expected, the high reactivity of **3** can be attributed to its good electron donor properties and its low steric hindrance around the Ge–Ge bond [56].

From electrochemical measurements of **23**, **26** and **27**, both oxidation and reduction processes were found to be irreversible. Figure 5 shows that the *E*°^x^_1_ values of **23**, **26**, and **27** shifted to more negative potentials by 530 to 610 mV, compared with that of pristine Lu_3_N@*I_h_*-C_80_. Cathodic shifts of *E*^red^_1_ values were also observed for **23**, **26**, and **27** within the range of 120 to 220 mV. The *E*°^x^_1_ and *E*^red^_1_ values of **27** showed slight cathodic and anodic shifts, respectively, compared to the corresponding redox potentials of **23** and **26**. As a result, **27** was found to be oxidized and reduced more readily than **23** or **26**. These cathodic shifts of *E*°^x^_1_ values of **23**, **26** and **27** were confirmed to be remarkably larger than those of **16a** and **16b**, as expected from the number of silicon and germanium atoms introduced into the fullerene cages.

## 9. Reactions of Sc_3_N@*I_h_*-C_80_ with Silirane 28

**Scheme 8:**
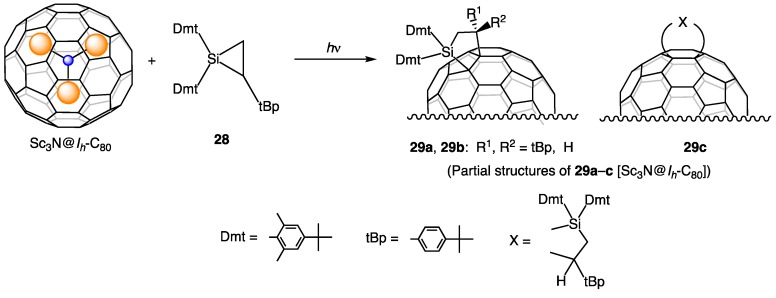
Synthesis of silirane adducts of Sc_3_N@*I_h_*-C_80_.

As described above, the addition reaction between Sc_3_N@*I_h_*-C_80_ and silylene produced the corresponding adduct in poor yield. This result prompted us to develop an alternative silylation reaction of Sc_3_N@*I_h_*-C_80_ using silirane **28** [57]. Photoirradiation of a toluene solution of Sc_3_N@*I_h_*-C_80_ and **28** afforded three compounds **29a**, **29b**, and **29c** (Scheme 8). Results of MALDI-TOF mass spectrometry, VIS-NIR and NMR spectroscopy suggest that **29a** and **29b** are a pair of diastereomers of [5,6]-adducts, whereas **29c** might be a [6,6]-adduct. However, further characterization of **29c** has not yet been achieved because of its low yield. It is noteworthy that the carbosilylation of La_2_@*I_h_*-C_80_ using **8** was conducted under thermal conditions. However, the reaction of La_2_@*I_h_*-C_80_ using **8** did not proceed efficiently under photolytic conditions [43].

**Figure 7:**
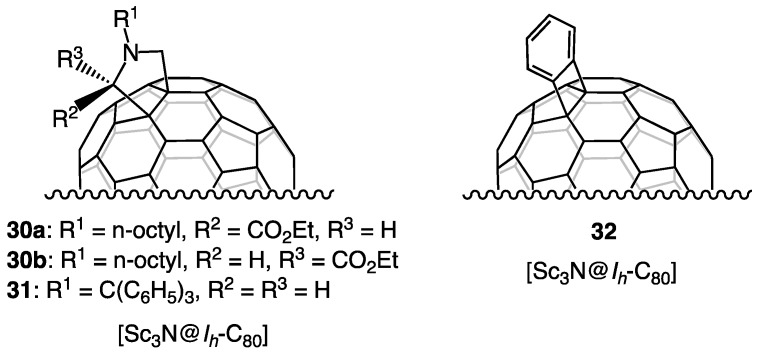
Partial structures of **30a**, **30b**, **31** and **32**.

The *E*°^x^_1_ values of **29a** and **29b** shifted toward more negative potentials by 380 and 370 mV, respectively, relative to that of Sc_3_N@*I_h_*-C_80_ (Figure 4). However, the *E*^red^_1_ values of **29a** and **29b** were nearly equal to that of Sc_3_N@*I_h_*-C_80_. These properties were verified based on the HOMO and LUMO energies of **29a** and **29b** obtained using theoretical calculations. To confirm the electronic effects of carbosilylation, these values were evaluated by comparing with those of the [5,6]-pyrrolidino adducts **30a** [58], **30b** [58], and **31** [59,60], and the [5,6]-benzyne adduct **32** [61] (Figure 7). Results show that both the *E*°^x^_1_ and *E*^red^_1_ potentials of **29a** and **29b** were shifted slightly toward more negative values than those of **30a**, **30b**, **31** and **32**. By coupling the redox properties of **9a** and **9b**, it appears that carbosilylation of EMFs produces slight cathodic shifts of the redox potentials compared to those produced by derivatization with carbon-based addends.

## 10. Dynamic Behaviors of the Encapsulated Atoms

The behavior of encapsulated metal atoms is an interesting issue affecting chemical and physical properties of EMFs. The movements of encapsulated metal atoms are affected by both the electrostatic metal–cage and the metal–metal interactions. In the case of monometallic EMF, metal atoms tend not to move around inside the cages. In dimetallofullerenes with highly-symmetric carbon cages, such as M_2_@*I_h_*-C_80_, two atoms can rotate in every direction along the endohedral surface [62,63,64,65]. However, the positions and behaviors of metal atoms, as well as their electronic properties, were found to be altered by exohedral derivatization. For example, the encapsulated Ce atoms in **6** were observed to be fixed at two positions close to the six-membered ring on the equator of the *I_h_*-C_80_ cage via X-ray crystallographic and paramagnetic ^13^C-NMR spectral analysis [41]. In addition, variable temperature (VT) ^139^La NMR and X-ray crystallographic analysis of **5** revealed that the La atoms circulate two-dimensionally along the equator of the C_80_ cage [40]. These results were consistent with earlier reported results of theoretical calculations [66].

The dynamic behaviors of the encapsulated metal atoms in **9a** and **9b** were also studied using VT ^139^La NMR spectroscopy and theoretical calculations [43]. The results showed that the line width of the ^139^La signal is broadened at temperatures higher than 280 K, suggesting that the two La atoms move dynamically inside the carbon cage of **9a** and **9b**. Coupling of these results with the X-ray crystallographic analysis of **9a** suggests that the La atoms are moving inside an annular belt formed from the six-membered rings [43].

## 11. Potential Applications 

As shown in this review, various EMFs and empty fullerenes showed high reactivities toward disiliranes, which have activated Si–Si bonds. Moreover, polysilanes, i.e., polymers comprising of catenated Si–Si bonds as polymer backbones, have attracted considerable interest as novel functional materials because of their unique chemical and physical properties [67,68]. Based on the delocalization of σ–electrons along the backbones of polysilanes, they have been studied as future functional materials, such as conductive polymers [69,70,71,72,73,74,75,76,77,78], photoresists [79], and organic light emitting diodes (LEDs) [80]. Polysilanes are well-known for their high hole mobility, however, their photoinduced charge carrier generation is not efficient. Doping C_60_ as an acceptor into polysilanes resulted in photoinduced electron transfer between them, which dramatically improved the charge carrier generation efficiency [70,71]. The electron transfer processes between photoexcited C_60_ and polysilanes [81,82] or polygermanes [83] were confirmed by laser flash photolysis experiments.

Due to their photoconductive properties, polysilanes may be applied to novel organic semiconductor devices. Some researchers investigated the photovoltaic properties of bulk heterojunction solar cells using C_60_ and [6,6]-phenyl C_61_-butyric acid methyl ester (PCBM) as electron acceptors and polysilanes as electron donors [84,85,86,87]. Furthermore, composite materials composed of polysilanes and EMFs may exhibit improved electronic properties as compared with those of empty fullerene-based materials. The organic solar cells using Lu_3_N@*I_h_*-C_80_ as an acceptor material showed higher open circuit voltages and higher photovoltaic efficiencies than those of C_60_ because of the higher LUMO level of Lu_3_N@*I_h_*-C_80_ [48,49].

The MO levels of the silylated and germylated EMFs were remarkably altered by the electron donating silyl and germyl groups as compared with those of pristine EMFs. Therefore, silylation and germylation would be useful for adjusting the electronic properties of EMF derivatives in electronic devices. In addition, covalent bonding between fullerenes and polysilanes can be used to construct donor–acceptor conjugate structures. The first prototype polysilane that incorporated C_60_ into the polymer backbone was synthesized by the photoreaction of a polysilane with C_60_ [88,89,90,91]. Development of silylation and germylation reactions of fullerenes will provide useful methods for affording fullerene-polysilane/polygermane conjugates.

## 12. Summary

This review provided an overview of our studies of the synthesis and characterization of silylated and germylated derivatives of EMFs using active silicon compounds and germanium compounds. Among these, disilirane **1** has been used as a versatile substrate that is both photochemically and thermally reactive. A comparison of the redox potentials of EMFs revealed that the reactivities of EMFs toward **1** depend on the electron-accepting abilities of the EMFs. Moreover, NMR and X-ray crystallographic examinations revealed that silylation affects the dynamic behavior of the encapsulated metal atoms and clusters in the EMFs. For example, results show that the movement of the La atoms in La_2_@*I_h_*-C_80_ was restricted to two-dimensional rotation near the equatorial plane of the carbon cage upon addition of **1**. The electronic effects of silylation and germylation on EMFs were clarified by electrochemical examinations and theoretical calculations. In general, the redox potentials of silylated and germylated derivatives shifted to more negative values relative to those of pristine EMFs. In particular, the cathodic shifts of the oxidation potentials were found to be remarkably large, reflecting the electron-donating effects of the silyl and germyl groups. These results demonstrate that EMFs with novel physical properties and functionalities are obtainable through chemical modification by exploiting the properties of various heteroatom-based functional groups.
